# Study protocol for the translating research in elder care (TREC): building context – an organizational monitoring program in long-term care project (project one)

**DOI:** 10.1186/1748-5908-4-52

**Published:** 2009-08-11

**Authors:** Carole A Estabrooks, Janet E Squires, Greta G Cummings, Gary F Teare, Peter G Norton

**Affiliations:** 1 Faculty of Nursing, University of Alberta, Edmonton, Alberta, Canada; 2 Health Quality Council, Saskatoon, Saskatchewan, Canada; 3 School of Medicine, University of Saskatchewan, Saskatoon, Saskatchewan, Canada; 4 Faculty of Medicine, University of Calgary, Calgary, Alberta, Canada

## Abstract

**Background:**

While there is a growing awareness of the importance of organizational context (or the work environment/setting) to successful knowledge translation, and successful knowledge translation to better patient, provider (staff), and system outcomes, little empirical evidence supports these assumptions. Further, little is known about the factors that enhance knowledge translation and better outcomes in residential long-term care facilities, where care has been shown to be suboptimal. The project described in this protocol is one of the two main projects of the larger five-year Translating Research in Elder Care (TREC) program.

**Aims:**

The purpose of this project is to establish the magnitude of the effect of organizational context on knowledge translation, and subsequently on resident, staff (unregulated, regulated, and managerial) and system outcomes in long-term care facilities in the three Canadian Prairie Provinces (Alberta, Saskatchewan, Manitoba).

**Methods/Design:**

This study protocol describes the details of a multi-level – including provinces, regions, facilities, units within facilities, and individuals who receive care (residents) or work (staff) in facilities – and longitudinal (five-year) research project. A stratified random sample of 36 residential long-term care facilities (30 urban and 6 rural) from the Canadian Prairie Provinces will comprise the sample. Caregivers and care managers within these facilities will be asked to complete the TREC survey – a suite of survey instruments designed to assess organizational context and related factors hypothesized to be important to successful knowledge translation and to achieving better resident, staff, and system outcomes. Facility and unit level data will be collected using standardized data collection forms, and resident outcomes using the Resident Assessment Instrument-Minimum Data Set version 2.0 instrument. A variety of analytic techniques will be employed including descriptive analyses, psychometric analyses, multi-level modeling, and mixed-method analyses.

**Discussion:**

Three key challenging areas associated with conducting this project are discussed: sampling, participant recruitment, and sample retention; survey administration (with unregulated caregivers); and the provision of a stable set of study definitions to guide the project.

## Background

In this issue of Implementation Science we present a series of three study protocols: an overview of the Translating Research in Elder Care (TREC) program [1]; TREC project one (Study Protocol for Translating Research in Elder Care: Building Context – an Organizational Monitoring Program in Long-Term Care Project – this paper); and TREC project two (Study Protocol for Translating Research in Elder Care – Building Context through Case Studies in Long-Term Care Project) [2]. The purpose of this paper is to report the study protocol for project one.

Increasingly investigators recognize that theory is required to guide the design of knowledge translation studies [3-5]. Currently, there is no one accepted theory of knowledge translation. Numerous theories are used in the field, many arising from the fields of organizational behaviour and social sciences, suggesting that knowledge translation is concerned not only with the behaviour of individual clinicians but also with the organizations or contexts in which they work. Most of these theories are neither highly developed nor rigorously tested, indicating a need for further work in this area.

### Knowledge translation theory

Rogers' representation of classical Diffusion of Innovations theory [6] is the dominant and most consistently used theory in this field. In it, Rogers describes the spread of new ideas using four main elements: the innovation, time, communication channels, and a social system. In addition to theories, a range of models addressing more focused areas of knowledge translation are also available [4,7] (Table 1). A recent framework with similarities to Rogers' Diffusion of Innovations Theory, is the *Promoting Action on Research Implementation in Health Services *(PARiHS) framework [8]. Its authors argue that successful research implementation (a specialized form of knowledge translation) is a function of the interplay between evidence, context, and facilitation. They hypothesize that it is when each of these three elements is high that successful research implementation is most likely to occur [9-11].

**Table 1:** Knowledge translation models

**Research Utilization Models**
Ottawa Model of Research use [110]

Conduct and Utilization of Research in Nursing (CURN) [111]

Nursing Child Assessment Satellite Training (NCAST) [112]

Stetler Model [113]

Iowa Model of Research in Nursing Practice [114]

Promoting Action on Research Implementation in Health Services (PARiHS) [8]



**Weiss' (Social Sciences) Research Utilization Models**

Knowledge-Driven Model [115]

Problem-Solving Model [115]

Interactive Model [115]

Political Model [115]

Tactical Model [115]

Enlightenment Model [115]



**Organizational Innovation Models**

Model of Territorial Rights and Boundaries [116]

Dual Core Model of Innovation [117]

Ambidextrous Model [55]

Bandwagon Models [118]

Desperation-Reaction Model of Medical Diffusion [119]



**Organizational Models and Theories** (Less focused on knowledge translation but relevant to knowledge translation)

Episodic or Punctuated Equilibrium Model of Change [120]

Situated Change Theory [121]

Agency Theory [122]

Institutional Theory [123]

### Predictors of knowledge translation

Rogers [6] argued that the adoption of an innovation (or research) is influenced by the interaction among three key components: the innovation, the adopter, and the environment. Investigators studying nursing services delivery have used this theory widely to frame studies of research use [12-20]. Little work has been done on characteristics of the innovation in healthcare [21]. Until recently, research has focused largely on changing individual (the adopter) behaviour. For example, in studying physician behaviour, investigators have focused on interventions, such as academic detailing [22], educational influentials [23-25], reminder systems [22,26], and audit and feedback [27,28]. While these interventions result in modest to moderate improvements in patient care, generalizability remains uncertain because of a limited understanding of the contextual, individual, and organizational factors that may influence the effectiveness of the different interventions [25,29].

In the study of nurse (adopter) behaviour, the focus has largely been on examining individual determinants of research use, such as attitude [30-32], age [31,33], education [17,33-36], experience [31,33], clinical area [17,30], journals read [19,37,38], employment status [33], and most recently, critical thinking behaviour [39]. Less attention has been given to interventions, such as opinion leaders [34] or multidisciplinary teams [40]. In a systematic review by Estabrooks *et al*. [41], the most frequently studied individual determinant, and the only one with a consistently positive effect, was attitude towards research. Findings for other individual determinants were highly equivocal and most studies were characterized by serious design and methodological flaws. Further, investigators have not selected individual factors for study with the important requirement that the factor be potentially modifiable.

Numerous organizational (environmental) factors thought to influence innovation adoption in industry and health services have also been studied. Those shown to have an influence include organizational complexity [42-46], centralization [47], size (*e.g*., number of beds) [20,42,44,48,49] presence of a research champion [50-52], traditionalism [53,54], organizational slack [42,55], access to and amount of resources [56], constraints on time [12,57-67], professional autonomy [58,68,69] and organizational support [30,31,56,68,70,71]. Again, investigators have generally not selected factors for study with a requirement for potential modifiability.

While there is generally a growing awareness and acceptance among researchers of the importance of organizational context (the local environment) to successful knowledge translation, and successful knowledge translation to improved patient, provider (staff), and system outcomes, astonishingly little empirical evidence supports these assumptions. Further, we know little about knowledge translation in the long-term care environment – an environment where: the quality of care is suboptimal [72] and the model of care is a nursing services delivery model where the majority of caregivers provide some level of nursing services.

In this project, we aim to investigate the impact of organizational context (giving specific attention to those factors which may be potentially modifiable) on knowledge translation and the effect of both organizational context and knowledge translation on resident, provider (staff), and system outcomes using long-term care as a naturally occurring laboratory.

### Theoretical framing

We are using an extension of the PARiHS framework to frame this research project. In the PARiHS framework, the continuous interaction between context, evidence, and facilitation is hypothesized to lead to increased research implementation. This project is particularly focused on increasing understanding of the role of one of these elements, context, on promoting knowledge translation and improving outcomes. We define context as "...the environment or setting in which people receive healthcare services, or in the context of getting research evidence into practice, the environment or setting in which the proposed change is to be implemented." [[73], p. 176]. Context according to PARiHS consists of three core dimensions: culture, leadership, and evaluation. In this project, however, we take an expanded view of context to include additional modifiable elements of the work setting, such as interactions (formal and informal), social capital, resources, and organizational slack.

### Study purpose and objectives

The purpose of this project is to establish the magnitude of the effect of organizational context on knowledge translation, and of organizational context and knowledge translation on resident, provider (staff), and system outcomes. The primary objectives of the project are:

1. To develop and validate theory relating to knowledge translation and its relationship to outcomes.

2. To develop and run an organizational monitoring system to assess organizational context in long-term care facilities longitudinally.

3. To measure the influence of organizational context on knowledge translation, and on resident, provider (staff), and system outcomes.

4. To undertake and complete multi-level modeling and mixed-method analyses.

5. To refine the TREC survey (a survey suite) to ensure it enables valid longitudinal measurement of organizational context in long-term care settings.

### Design and methods

#### Design

This project is a multi-level, longitudinal descriptive study of a stratified random sample of long-term care facilities across the three Canadian Prairie Provinces: Alberta, Saskatchewan, and Manitoba. Data are collected at three levels: facility, unit, and individual (provider [staff] and resident). Facility-level data are collected annually from facility administrators and unit level data, quarterly from care managers. Provider (staff)-level data are collected annually from unregulated staff (*i.e*., healthcare aides), regulated staff (*i.e*., licensed practical nurses/registered nurses, physicians, allied healthcare providers, practice specialists [*e.g*., educators, advanced practice nurses]), and managerial staff (*i.e*., unit care managers) using the TREC survey. Resident-level data are accessed quarterly from the Resident Assessment Instrument-Minimum Data Set version 2.0 (RAI-MDS 2.0) databases that are maintained by provincial, regional, and/or facility custodians (depending on the province).

### Measures

#### Facility- and unit-level measures

Standardized data collection forms, developed by the research team in consultation with TREC senior decision makers, are used to collect unit- and facility-level data. Examples of data collected using these forms include: facility operation model (*e.g*., public, private, voluntary), facility structure (*e.g*., number and type of units), services/programs offered (at unit and facility level), major events, and staffing patterns.

#### Provider (staff)-level measures

The TREC survey is used to collect provider (staff)-level data. The survey is composed of a suite of survey instruments designed to measure: organizational context, knowledge translation, individual factors believed to impact knowledge translation, and staff outcomes believed to be sensitive to both organizational context and knowledge translation. The core of the TREC survey is the Alberta Context Tool (ACT), a survey designed to measure organizational context in complex healthcare settings. The index version of the ACT was developed for use in acute care settings [74] and has been adapted for and piloted in the long**-**term care setting as part of our feasibility work for this project. There are variations of the tool for each of the following groups: healthcare aides, nurses (licensed practical nurses/registered nurses), physicians, allied healthcare providers, practice specialists, and care managers. In addition to the ACT, several additional scales are included in the TREC survey. They include: self-reported knowledge translation (operationalized as the use of research or best practice); individual factors – attitude towards research use, belief suspension, and problem solving ability; and measures of staff outcomes – burnout, aggression from residents, job and career satisfaction, and health status.

### Psychometric properties of the TREC survey

#### The ACT

The ACT is a 51-item measure of organizational context. The tool includes eight dimensions: leadership, culture, evaluation, formal interactions, informal interactions, social capital, structural and electronic resources, and organizational slack. The first three dimensions assess organizational context as conceptualized in the PARiHS framework [8], while dimensions four through eight represent our expanded view of organizational context. Taken together, these eight dimensions, using principal components analysis, have revealed a fourteen-factor structure explaining 70% of the variance in organizational context in acute care (hospital) settings. Further, in the acute care sector each dimension has shown acceptable internal reliability (Cronbach α, range = 0.65 to 0.92) [74]. While initial psychometric analyses from our long-term care feasibility work were limited by sample size, we have been able to verify a stable three-factor structure representing 74% of the variance in organizational context for the first three dimensions of the ACT (leadership, culture, and evaluation) in long-term care. Reliability coefficients (Cronbach α) for the eight dimensions were acceptable.

#### Knowledge translation

Knowledge translation, in the TREC Survey, refers to the use of research or new knowledge in practice. Four types of research utilization (instrumental, conceptual, persuasive, and overall) are assessed. The items used to measure research use have produced consistent findings in past studies [75,76] indicating reliability. Construct validity of the measures with structural equation modeling has also been reported [77].

#### Attitude

Attitude, in the TREC survey, refers to the opinion expressed, along a continuum of negative to positive, by healthcare workers towards research knowledge. A six-item abbreviated scale is used based on Lacey's [78] modification of a questionnaire developed by Champion and Leach [31]. The abbreviated scale has demonstrated good reliability (Cronbach α = 0.74) and construct validity (one factor accounting for 48% of the variance in 'attitude towards research') [79].

#### Belief suspension

Belief suspension refers to the degree to which an individual is able to suspend previously held beliefs in order to implement a research-based change. It measures personal beliefs of the healthcare worker (*i.e*., those beliefs that originate in the family of origin [the home], in school/training, or within the work context). A six-item scale (three items measuring willingness to suspend belief, and three items measuring actual suspension of belief) developed by Estabrooks [80] is used in the TREC survey. The scale has shown good reliability (Cronbach α = 0.87) and construct validity (two factors accounting for 78% of the variance in 'belief') in previous research [80].

#### Problem-solving ability

Problem-solving ability refers to the ability of an individual to implement behaviors that reflect a goal directed sequence of cognitive operations utilized to cope with challenges or demands [81]. An abbreviated form (10 items) of Heppner's 32-item Problem Solving Inventory (PSI) is used in the TREC survey. The abbreviated form has shown good reliability (Cronbach α = 0.74) and construct validity (three factors corresponding to the original three factors of the 32-item PSI, accounting for 61% of the variance in 'problem solving ability') [80]. In this project, we have permission to append the abbreviated version to the TREC survey.

#### Burnout

Burnout is assessed using the Maslach Burnout Inventory General Survey (MBI-GS) [82,83]. In this instrument, respondents are asked to indicate the frequency with which they have experienced specific feelings. The original MBI-GS contained 16 items, and is reliable with Cronbach α coefficients ranging from 0.88 to 0.90 for its subscales [83,84]. Factorial validity using structural equation modeling and construct validity based on convergence and divergence have also been reported [84]. In this project, we have permission to append the MBI-GS (short-form), which consists of nine items, to the TREC survey.

#### Health status

Health status is measured using the SF**-**8™ Health Survey, a multi-purpose short**-**form health survey with eight questions. It yields an eight**-**scale profile of functional health and well**-**being scores, as well as psychometrically-based physical and mental health summary measures and a preference**-**based health utility index. The eight questions included in the SF**-**8™ Health Survey were selected from pools of empirically tested items, and are scored on the same norm-based metric as the original larger SF-36 scale [85]. Items in the SF**-**8™ Health Survey ask respondents to consider a specific period of time, or recall period, when responding. The instrument has shown good reliability (Cronbach α coefficients of >0.76 for all eight subscales, and a test**-**retest reliability coefficient of >0.80) [85]. Construct validity using factor analysis has also been established [85]. We have permission to append the standard form (four-week recall) of the SF**-**8™ Health Survey to the TREC survey.

#### Aggression in the workplace

Aggression in the workplace is measured in the TREC survey with a modification of the Workplace Violence Instrument (WVI). The WVI consists of a subset of questions developed by Estabrooks and colleagues [86] based on a critical review of the literature and is designed to assess six types of aggressive (violent) behavior: inappropriate yelling or screaming; verbal threats; hurtful remarks or behaviors; spit on, bitten, hit, pushed or pinched; repeated and unwanted questions or remarks of a sexual nature; and sexual touching. The scale has shown variation in a large international study (indicating reliability) [87,88].

#### Resident-level measures

Resident demographic and outcome data are collected (retrospectively) at the unit and facility level (that is, de-identified at the individual resident level) using routinely collected RAI-MDS 2.0 data. The RAI**-**MDS 2.0 is an international system for capturing essential information about the health, physical, mental, and functional status of continuing and long-term care facility residents [89-97]. It consists of seven assessment modules and tracking forms, including an initial or admission assessment, annual assessment, quarterly assessments, assessments for major health-related events, as well demographic change, discharge, and facility profile tracking forms. The instrument is used in long-term care facilities across the Prairie Provinces where this project is taking place. Numerous reports describe the reliability, validity, and sensitivity of change of the indicators of resident outcomes captured with the instrument [96,98-104]. In this project we are initially focusing on the following four indicators as outcome variables in our analysis: pain management, falls and fractures, problem behavior management, and the health status index – a composite measure of health-related quality of life. During the five years of the project other resident outcomes captured with the RAI-MDS 2.0 data may also be used.

### Procedures (year one)

#### Feasibility testing and piloting of the TREC survey

Investigation of knowledge uptake in the long-term care sector is nascent. Therefore, our first year's work was to undertake feasibility testing in the sector and pilot the TREC survey in long**-**term care facilities with frontline workers. The purpose of this feasibility work has been to: tailor the TREC survey for use by frontline (primarily healthcare aide) workers in the long-term care environment; assess the feasibility of our data collection procedures and modify them accordingly for the main project; and confirm/establish reliability and validity of the survey in the long-term care context.

We conducted feasibility and pilot testing of the TREC survey with unregulated, regulated, and managerial staff in all three Prairie Provinces. Our pilot work demonstrated that online surveys were not a viable option for the healthcare aide group at this time, and that the survey could be administered more effectively and in a shorter time interval with these workers by using a structured interview format (mean time using personal interview of 19 minutes compared to a mean time using pen and paper of 35 minutes). Therefore, we are using a computer-assisted personal interview (CAPI) format of survey administration with healthcare aide staff in the main project. Based on acceptable response rates with online versions of the ACT in acute care settings with regulated and managerial workers [74,105] we are offering the TREC survey in online format only to these groups.

### Sampling

#### Facility sample

Our sample consists of two facility (*i.e*., nursing home) samples. Our primary sample consists of urban facilities drawn proportionately from the three provinces. We require a minimum of 25 facilities for multi**-**level modeling [106]. We have therefore over**-**sampled (to 30 urban facilities) to account for facility attrition over the five-year period and to strengthen our models. A second sample is composed of rural facilities. We realize that care in rural settings may present different challenges and opportunities from those in urban settings. Therefore, we are studying six rural facilities in our sample. All rural facilities are located within the province of Saskatchewan as they deliver more care in rural settings than the other Prairie Provinces. Thus, our combined facility sample size is 36 facilities.

Facility selection in the urban facility sample is by stratified random sampling with replacement. All long-term care facilities in the three Prairie Provinces meeting our inclusion criteria (Table 2) have been stratified by healthcare region (within province), operational model (public, private, voluntary) and size (small: 35 to 149 beds, large: = 150 beds) resulting in the generation of six facility lists per region: public small, voluntary small, private small, public large, voluntary large, and private large. We have stratified based on size because previous organizational innovation literature strongly indicates it is associated with innovation [47]; our decision-maker partners agree that size is an important dimension in this study. We have also stratified based on owner-operator model because our decision-maker partners argued strongly that it is an important factor in assessing context, knowledge translation, and resident outcomes. The three types of owner-operator models reflect those found in the three participating provinces. Each stratified list was shuffled using a random number generator to create final lists of selected facilities by province. These lists are held by the provincial lead investigators who follow a standardized procedure for recruitment, and if needed, replacement of facilities. A similar sampling strategy was used to select the six rural facilities.

**Table 2:** Facility Inclusion and Exclusion Criteria

**Facility Inclusion Criteria**	1. Registered by the provincial government
	2. 90% of residents over 65
	3. Conduct RAI-MDS 2.0 assessment since September 2007
	4. Facility operation conducted in the English language
	5. Rural sites greater than 100 km (but less than 200 km) radius of Regina or Saskatoon, and with populations of 10,000 people or less
	6. Urban facilities must be within designated health regions (i.e., Alberta – Edmonton, Calgary, or East Central; Manitoba – Winnipeg; Saskatchewan – Regina-Qu'Appelle or Saskatoon)
	7. Stable or minimal level of organizational flux
**Facility Exclusion Criteria**	1. Facilities integrated with acute care
	2. Facilities with a sub-acute service
	3. Rural facilities within the Capital Health Region (Edmonton, AB), Calgary Health Region (Calgary, AB), and Winnipeg Regional Health Authority (Winnipeg, MB) that reside in places with populations of 10,000 people or less
	4. Rural facilities less than 100 km or greater than 200 km of Regina or Saskatoon (SK)
	5. Facilities with less than 35 long-term care beds
	6. Dementia special needs facilities
	7. Facilities undergoing (or expected to undergo) a degree of organizational flux within the proposed five-year lifespan of the TREC program

#### Provider (staff) sample

Participants are recruited using a volunteer, census-like sampling technique. All healthcare aides, regulated and managerial staff in the 36 long-term care facilities who meet our inclusion criteria (Table 3) and can be contacted (*i.e*., personally or through mail) are invited to participate.

**Table 3:** Provider (Staff) Inclusion and Exclusion Criteria

**Healthcare Aides**	**Inclusion Criteria:**
	1. Identify a unit within a facility where they have worked for at least three months and are working now
	2. Work a minimum of six shifts per month on this unit
	**Exclusion Criteria:**
	1. Healthcare Aide Student
**Nurses**	**Inclusion Criteria:**
(Registered Nurses [RNs] and Licensed Practical Nurses [LPNs])	1. Identify a unit within a facility where they have worked for at least three months and are now working
	2. Work a minimum of six shifts per month on this unit
	**Exclusion Criteria:**
	1. Licensed Practical Nurse/Registered Nurse Student
	2. Nursing instructors whose primary role is supervising students

**Allied Healthcare Providers**	**Inclusion Criteria:**
	1. Identify a facility in which they provide at least one third (*i*. *e*., at least 6 days a month) of their long-term care services
	**Exclusion Criteria:**
	1. Allied Healthcare Student
	2. Allied instructors whose primary role is supervising students

**Physicians**	**Inclusion Criteria:**
	1. Physicians who see ten or more residents in a facility
	2. The Medical Director of the facility
	**Exclusion Criteria:**
	1. Physicians not currently seeing residents
	2. Residents or medical students
	3. Academic staff

**Practice Specialists**	**Inclusion Criteria:**
	1. Identify a facility in which they provide at least one third (*i*. *e*., at least six days a month) of their long-term care services
	**Exclusion Criteria:**
	1. Academic staff
	2. Clinical instructors whose primary role is supervising students

**Care Managers**	**Inclusion Criteria:**
	1. Identify one facility in which they work more than 50% of the time.
	2. Facility administrators when there is no care manager who is responsible for resident care (*e*. *g*. only one unit in the facility)
	**Exclusion Criteria:**
	1. Managers **not **responsible for resident care (*e*. *g*., dietary managers, materials management managers)

We will aggregate the healthcare aides' scores on the TREC survey to compute unit and facility scores; healthcare aides are the primary care providers for residents and provide the majority of direct nursing and related services to residents in long-term care facilities. Based on our previous work with the ACT (and using a two-sample mean sample size calculation), we estimate needing a minimum of ten healthcare aides per unit to complete the TREC survey in order to get stable estimates for aggregated unit scores on the survey's constructs. This is consistent with previous work that we have completed [107,108].

### Procedures (years two to five)

#### Data collection

Each province has established a local team responsible for recruitment and data collection. This team is led by a site investigator(s) and includes a research manager, research associate, research assistant(s), and in some cases graduate students and post-doctoral fellows.

#### Facility and unit level data

We are collecting facility-level data (*e.g*., funding, resident census, staffing, services and programs, and staff absence) using standardized data collection forms which are administered in short structured interviews with facility administrators (directors of care). Stable items (*e.g*., postal code, age of facility) are being collected only at the start of the project. Other items (*e.g*., major events, staff turnover) are collected for each year of TREC survey data collection. We are also collecting unit-level data (*e.g*., type of unit, average length of resident stay, number of occupied beds, staffing patterns) using standardized unit data collection forms. These are also administered for each year of TREC survey data collection in short structured interviews with unit care managers.

#### Provider (staff)-level data

Members of each provincial research team, in consultation with the site administrator (or designate), arrange for recruitment of study participants. Potential participants are informed about the study through a variety of communication strategies, including informal information sessions in each facility by a member(s) of the local research team. Potential participants are provided with a study information sheet at this time.

Staff in the 36 facilities are asked to complete the TREC survey. The survey contains 141 to 167 items, depending on the target staff group. A vendor [109] has been contracted to develop and administer the electronic/online version of the survey (for the regulated and managerial staff) and to develop the CAPI version of the survey (for the healthcare aides). In both administration methods the vendor is responsible for secure, accurate, and reliable data capture with appropriate linkages, and secure transfer of the data to the central study server.

Interviewers (trained TREC research staff and contracted interviewers) administer the CAPI survey to healthcare aides. The interviews are completed during the healthcare aide's work time, or if they prefer, an alternative time and place is arranged. Interviewers are trained in both technical aspects of the CAPI process as well as interview technique and trouble shooting. Quality control practices specific to the CAPI interviewing are in place and will be monitored and maintained through the duration of the project.

For the online surveys, a survey package containing an information letter/invitation to participate is distributed by a member of the research team to all regulated and managerial workers in the selected facilities that meet our inclusion criteria and can be contacted. This survey package contains a business card with the URL and a password to enable access to the survey. In addition, the package contains a coffee card as a token of our appreciation and information sheets. There is no opportunity for the participant to identify themselves to the research team. Completed web surveys will not contain names or identifying information. Further, the computer data will be password protected and only accessible to the research team working on this study. Two weeks and four weeks following the distribution of initial survey packages, a printed reminder (in the form of a poster) is posted on the units of the participating facilities.

#### Resident-level data

RAI-MDS 2.0 data are collected, in electronic format, on a quarterly basis as part of routine clinical care at all of the long-term care facilities in the health regions involved in this project. Staff in the central data processing unit for TREC (located at the University of Alberta) are responsible for receiving and managing the RAI**-**MDS 2.0 data (in electronic format) from the appropriate provincial/facility custodians on a quarterly basis for the duration of the project. The data are supplied de-identified at the level of the individual resident but contains (or they can be created) unit- and facility-level identifiers (needed to conduct our multi-level modeling).

### Data quality

Interviewer training for individuals conducting CAPI with healthcare aides has been undertaken to ensure standardized interviewer technique and the collection of high-quality data. Interviewer and quality control manuals have been created to facilitate data quality processes for these interviews. The interviewer manual describes the step-by-step process of conducting a CAPI interview, and the process by which the data are handled. The quality control manual outlines the characteristics of a successful interviewer and the training and process that must be undertaken before someone is deemed to be prepared to begin interviewing. Quarterly and yearly quality control and improvement processes are in place.

### Data analysis

Data analysis is an ongoing iterative process. Data are cleaned and processed for analyses at the close of each quarter. Real-time descriptive analyses are completed more frequently to assess response rates and to ensure that interviewer variation is within expected limits. As the data set is assembled, we are performing ongoing descriptive analyses to: check for outliers and systematic biases, monitor response rates, and inform variable selection for modeling. These analyses are also being used to inform TREC project two data collection [2]. In addition, we are computing response rates and distributions (means, medians, standard deviations) for the knowledge translation measures and all of the constructs assessed in the TREC survey by provider group, unit, facility, region, and province.

### Psychometric analysis (ACT)

Psychometric analyses on the ACT component of the TREC survey will be carried out to determine the tool's robustness in the long-term care setting (pilot testing in the LTC setting yielded satisfactory results). In brief, we will conduct reliability (internal consistency) and validity (factor analysis, item analysis, and modeling) analyses. We will examine corrected item-total correlations and coefficient alpha. Exploratory factor analytic methods will be used to: indicate the underlying domains (factors) within the item pool of the ACT, which will provide an explanation of variance amongst items; to operationalize the meaning of the underlying factors; and to determine if our derived variables (*e.g*., organizational slack) behave as expected. Construct validity assessments with confirmatory factor analysis (using structural equation modeling) will also be performed.

### Multi-level modeling

After reviewing the descriptive analyses for the total data set we will undertake analysis of variance (ANOVA) and multiple comparison tests as sample size permits in order to investigate differences in knowledge translation behaviours among staff groups (*i.e*., healthcare aides, nurses, physicians, allied health, practice specialists, care managers) and between units, facilities, regions, and provinces. We will use similar methods to describe and assess differences in resident (*e.g*., falls) and provider (*e.g*., health status, burnout) outcomes. Additionally, differences among staff groups, units, facilities, regions, and provinces on all independent variables (*e.g*., ACT dimensions) will be examined with similar descriptive and ANOVA methods.

The majority of our analytical work will consist of a series of regression models, then multi-level and structural equation models, and finally, if our data permits, hierarchical structural equation models. We will estimate the knowledge translation dependent variables at the individual provider (staff) level. Staff characteristics, individual, and context variables will be the primary explanatory variables in these equations. We will then use the predictions of knowledge translation variables as independent variables in additional equations to estimate staff and resident outcomes, with the individual staff member/resident as the unit of analysis. Resident characteristics, staff characteristics, and predictions of knowledge translation variables aggregated to the unit or facility level will be the primary explanatory variables in these equations. We will perform multi-level analysis using organizational data (aggregated) at the unit level with subjects nested within each unit. We have three levels of organizational data in the survey- facility (level three), unit (level two), and individual (level one). In further analyses, we will use these data in structural equation models to explore the relationships among different outcomes and context variables, including latent variables. This may be of particular importance in analyzing the knowledge translation variables, which have qualities that are difficult to observe directly.

### Facility reports

As a value-added function for the participating long-term care facilities we will provide them with annual facility reports approximately six weeks after we have the second wave of data collection (so that we can provide wave 1 and wave 2 comparisons). Our decision makers have informed us that because many of the long-term care facilities within their regions have limited internal data analysis capability, periodic private reports on their own data would be of value. These reports will be at the facility level and may include some de-identified unit-level feedback, but will not allow for identification of specific residents, staff and/or units. The format of the report will be the same in all facilities and have been determined in a consultative process with the facilities in the first year of the main study. In addition to the agreed upon data elements requested by the facilities, a section of the report will also emphasize variances of note for the individual facility.

### Feedback to Health Care Aides

Our original intention was to disseminate survey results at the end of the 5-year program. However, during year one HCA's voiced a strong desire to receive feedback as the study progressed. Consistent with the *integrated KT *approach we are using and in response to this request, a decision was made to provide feedback to HCA's following each wave of TREC survey data collection. To this end, we developed feedback reports and established a process to evaluate their effectiveness. The report development phase involved selection of single items from the survey, analysis of the data for the purposes of presenting comparative data, and preparation of sample feedback reports. We consulted with key stakeholders to elicit feedback on the sample reports; this informed a number of revisions to the reports. This feedback occurs shortly after the current wave of data collection is completed in a facility.

### Ethical review

Ethical approval for this project was obtained from the appropriate university ethics boards: Universities of Alberta, Calgary, Manitoba, Saskatchewan, and Regina. We have also received relevant operational approvals from the 36 selected long-term care facilities, as well as RAI-MDS 2.0 custodian approvals. Data collection has proceeded in quarters, occurring during all 12 months. All data in this study are held confidentially. Master files that can be linked to units and facilities are locked with restricted access. Other team members and staff will have access as required (*i.e*., for analysis) to data files with scrambled identification codes. All data are held centrally at the University of Alberta on secure dedicated servers according to Tri Council and generally accepted standards for similar data collections.

## Discussion

We anticipate that the proposed project, as one component of the larger TREC program, will contribute to the development of new knowledge translation theory about the role of organizational context in influencing knowledge use in long-term care settings (and particularly among unregulated caregivers), as well as the role of context on provider and resident outcomes.

There are a number of areas of challenge associated with this project. The first area of challenge relates to sampling, recruitment, and retention over the five-year period of the project. Our sampling approach was guided by the need to balance the selection of facilities by operation model, facility size, and province to the extent feasible. However, each province has differing numbers of facilities, as well as differing distributions of small and large facilities, and operation models. This has lead to some provinces being over- or under- represented in specific matrix cells.

Recruitment of staff participants has been challenging in our previous research. We are undertaking a comprehensive recruitment and retention process that began as we formulated the team and included senior decision-makers in each jurisdiction. Members of each provincial team visit the recruited facilities prior to commencing data collection and meet with all levels of staff to inform them of the study and its potential benefits. The project managers in each province maintain regular contact with each site and project co-lead investigators visit each province on a regular schedule. While we hope to maintain a stable number of respondents in each year of the project, we are not following a cohort of caregivers throughout the five years. While limiting some of our analytical possibilities, a cohort of staff is not necessary to examine the effect of context on knowledge translation, or staff and resident outcomes in the residential long-term care environment.

A second area of challenge for this project is survey administration, and in particular, administration to the healthcare aides. We had originally intended to use online surveys for all staff, including healthcare aides, although we knew we might need to use paper-based surveys for the healthcare aide group. Our early feasibility and pilot work demonstrated that online surveys were not a viable option for the healthcare aide group, at least at this time. We also discovered that traditional paper and pencil administration resulted in poor data quality. Therefore, we elected to administer the survey to this group using CAPI in the main project. Costs for this approach are higher than for the original, planned online survey administration. Therefore, analyses are planned to assess costs compared to benefits of using the CAPI approach. In these analyses, we will pay particular attention to the balance between data completeness, data quality and cost.

A third area of challenge relates to the provision of definitions to guide the project – as expected, we require standard definitions of terms to ensure consistency in data collection and analysis procedures between the three provinces. We have found, however, that a number of our definitions have required ongoing revisions. For example, we have found considerable variation between how a 'unit' is defined both between facilities in a province and between provinces. A standard definition of 'unit' that can be applied across settings is important to understanding the structure of different long-term care facilities, and also goes to aggregation of staff scores to create unit-level scores for the TREC survey constructs. With respect to creating standard definitions, another challenge we face is that different terms often are used to refer to the same concept between the three provinces. For example, in Alberta there are a several terms used to refer to unregulated workers (*e.g*., personal care attendant, healthcare aide, resident companion) that are different again from the term used in Saskatchewan (*e.g*., special care attendant). To address this definitional challenge, we are creating a universal TREC glossary that identifies all possible terms associated with the research program.

## Conclusion

The project described in this protocol will make an important contribution to the advancement of knowledge translation theory as far as it considers the effect of organizational context on knowledge translation, and the subsequent impact of organizational context and knowledge translation on resident and staff outcomes in residential long-term care settings. The theoretical insights will then be used to design interventions to modify elements of organizational context that improve knowledge translation and outcomes for residents and staff in future studies.

## Competing interests

The authors declare that they have no competing interests.

## Authors' contributions

CAE is the principal investigator for the TREC research program. She conceived the program and its design, secured its funding, is providing the leadership and coordination for the program, and provided substantial commentary to the final submitted manuscript. JES is a trainee within the TREC research program and made major contributions to drafting the study protocol and final manuscript. GGC, GFT, and PGN participated in designing the study, securing grant funding, and provided critical commentary to the final submitted manuscript. PGN is the co-lead investigator (with CAE) for the project described in this manuscript. All authors read and approved the final submitted manuscript.
